# Enhancing clinical complete response assessment in rectal cancer: integrating transanal multipoint full-layer puncture biopsy criteria: a systematic review

**DOI:** 10.3389/fonc.2024.1428583

**Published:** 2024-12-20

**Authors:** Xin Liu, Boshi Duan, Ruibin Liu, Mengying Zhu, Guohua Zhao, Ning Guan, Yue Wang

**Affiliations:** ^1^ Department of Colorectal Surgery, Cancer Hospital of China Medical University, Liaoning Cancer Hospital & Institute, Shenyang, China; ^2^ Department of Medical Oncology, National Cancer Center/National Clinical Research Center for Cancer/Cancer Hospital & Shenzhen Hospital, Chinese Academy of Medical Sciences and Peking Union Medical College, Shenzhen, China; ^3^ Department of Clinical Integration of Traditional Chinese and Western Medicine, Liaoning University of Traditional Chinese Medicine, Shenyang, China; ^4^ Department of Generall Surgery, Cancer Hospital of China Medical University, Liaoning Cancer Hospital & Institute, Shenyang, China; ^5^ Center of Medical Examination, Cancer Hospital of China Medical University, Liaoning Cancer Hospital & Institute, Shenyang, China

**Keywords:** Rectal Neoplasms, Neoadjuvant therapy, clinical complete response, watch and wait strategy, local resection

## Abstract

There is currently a lack of standardized criteria for evaluating clinical complete response (cCR) in rectal cancer post-neoadjuvant chemoradiotherapy (nCRT), often resulting in discrepancies with true pathological complete response (pCR). Staging local lesions via MRI is challenged by tissue edema and fibrosis post-nCRT, while endoscopic biopsy accuracy is compromised by residual cancer foci in the muscular layer. Transanal local excision offers a relatively accurate assessment of lesion regression but poses challenges including impaired anal function and elevated complication rates. Building on current diagnostic frameworks, we propose enhancing cCR assessment by integrating histological criteria from transanal multipoint full-layer puncture biopsy (TMFP). This approach aims to improve accuracy while minimizing complications, offering promise for patients opting for observation-based treatments. Further research is needed for definitive conclusions.

## 1 Introduction

Neoadjuvant chemoradiotherapy (nCRT) represents a prevalent therapeutic approach in cancer management, entailing the administration of chemotherapy and radiation therapy before surgery (1). Its primary objective is to diminish tumor size and heighten the likelihood of successful surgical resection (2). Conversely, watchful waiting stands as a conservative regimen for rectal cancer patients who achieve cCR (3). Post-nCRT, certain patients encounter either complete tumor disappearance or significant reduction, denoted as complete clinical response. Conventionally, these individuals would undergo standard total mesorectal excision surgery (4). However, emerging evidence suggests that surgical intervention in such cases may not consistently yield apparent survival benefits and could potentially instigate postoperative complications (5). For instance, Habr-Gama et al. (7) demonstrated that upfront combined chemoradiotherapy yielded manageable side effects. The TRG system was widely used after neoadjuvant chemotherapy to treat gastrointestinal cancers, with TRG 1a defined as the complete regression of tumor without residual cancer cells (6). In this study, approximately 30.5% of patients attained complete tumor regression within a median follow-up period of 36 months. The approach spared 26.2% of patients surgery, and 38.1% of cases that otherwise would have required a resection of the abdominoperineal sphincter were managed sphincter-saving (7).

Research indicates that patients undergoing watchful waiting do not exhibit a significant decrease in tumor-specific mortality, overall survival, or disease-free survival rates. Smith et al. (8) reported that almost eighty percent of patients in the watchful waiting group (WW) were able to preserve their rectal health after five years. The overall survival rate in the WW group was 73%, while the overall survival rate in the pCR group was 94%. Additionally, Dossa et al. (9) identified 23 studies involving 867 patients, with a median follow-up period of 12-68 months. They observed no notable distinction in outcomes between patients subjected to observation and those with clinical complete response who underwent surgery, in terms of non-regrowth recurrence (RR=0.58), cancer-specific mortality (RR=0.58), disease-free survival (HR=0.56), or overall survival (HR=3.91).

Despite achieving cCR post-nCRT, rectal cancer patients do not necessarily attain pCR. Dossa et al. (3) reported a pooled 2-year local regrowth rate of 15.7%, with 95.4% of regrowth cases receiving salvage therapies. Renehan et al. (10) noted that 129 patients managed by watchful waiting had local regrowth, of which 36 patients were salvaged (88%) when the regrowth was non-metastatic. van der Valk et al. (11) demonstrated out of 880 patients, 25.2% experienced local regrowth after 2 years, with 88% of cases detected within 24 months, and 97% manifesting inside the bowel wall. Distant metastases were observed in 71 (8%) cases. Consequently, a substantial proportion of patients achieving cCR post-nCRT do not attain pCR, with a notable incidence of local tumor regrowth and concurrent distant metastasis (11). This finding underscores concerns and uncertainties regarding the adoption of a watchful waiting strategy (12). While it may serve as a measure to avoid overtreatment, more proactive therapeutic interventions or closer monitoring may be warranted for these patients (13). Thus, treatment decisions for those achieving cCR after nCRT necessitate careful consideration to mitigate the risk of recurrence and metastasis, thereby enhancing patient survival (14).

Patients experiencing local regrowth during the watchful waiting period can undergo salvage surgery, often achieving a high rate of R0 resection (15). However, the recurrence of tumors during this period suggests that the tumors had already progressed to a more advanced stage before treatment, leading to a diminished overall survival rate postoperatively. Chadi et al. (15) demonstrated a 2-year cumulative incidence of local regrowth at 21.4%. Similarly, Cotti et al. (16) observed local regrowth was observed, with mesorectal fascia involvement occurring more frequently in this group (35.0% compared to 13.3%) in 20 patients (29.9%) following a watch-and-wait strategy. Salvage surgery was performed for all regrowth cases, with the majority (75%) undergoing sphincter-sparing procedures. The 5-year overall survival rate among patients with regrowth was 71.1%, in contrast to 91.1% among those maintaining sustained complete clinical response.

## 2 Current criteria for cCR assessment

(1) Consensus of Experts on Watch-and-Wait Strategy for Rectal Cancer After Neoadjuvant Therapy (2024 edition) in China (17):

The clinical diagnosis of cCR includes digital rectal examination, endoscopy, rectal magnetic resonance imaging (T2WI/DWI), pathological biopsy, thoracoabdominal-pelvic CT, rectal ultrasound, and carcinoembryonic antigen (CEA) levels. Patients with satisfactory tumor regression after neoadjuvant therapy but not meeting strict cCR criteria are considered to be in near-cCR.

(2) MSKCC Criteria from the United States (18):

The standards proposed by the Memorial Sloan Kettering Cancer Center (MSKCC) are widely used to assess the complete clinical response of rectal cancer patients. This criterion combines various methods such as rectoscopy, imaging evaluation, and pathological examination to determine whether patients have achieved complete clinical remission.

(3) European Society for Medical Oncology (ESMO) Guidelines (19):

The guidelines from the European Society for Medical Oncology (ESMO) provide a series of criteria for determining complete clinical remission. These criteria mainly include rectoscopy, imaging evaluation (such as MRI), biomarkers, and clinical symptoms. Complete clinical remission is considered when patients show disappearance or negativity in these aspects.

(4) Habr-Gama Criteria (20):

Proposed by Habr-Gama et al., this method evaluates the complete clinical remission of rectal cancer patients after neoadjuvant therapy. According to this criterion, patients must undergo a 12-week observation period after completing neoadjuvant therapy. During this period, if patients show negative results in rectoscopy, biomarkers, MRI examination, and biopsy, and no clinically palpable abnormalities, they are considered to have achieved complete clinical remission.

## 3 Current diagnostic methods

### 3.1 Colonoscopy biopsy:

For patients initially diagnosed with rectal tumors, colonoscopy biopsy pathology serves as a crucial diagnostic modality (21, 22). However, the accuracy of colonoscopy biopsy significantly diminishes following nCRT (23). While colonoscopy biopsy offers advantages such as simplicity, non-invasiveness, and repeatability, enabling the observation of morphological changes in lesions, its limitations, including restricted biopsy depth and low diagnostic accuracy, become pronounced (24, 25). Specifically, its limited accuracy compromises its utility in assessing cCR post-nCRT (26, 27). Consequently, one of the primary reasons many cCR assessment criteria overlook colonoscopy biopsy is due to its inherent limitations (26, 27). For instance, Duldulao et al. (28) observed that after neoadjuvant chemoradiation, cancer cells were detected in the mucosa of 12 individuals (13%) with ypT2-4 tumors, while 53 patients (56%) exhibited cancer cells in the submucosa; notably, cancer cells infiltrated the muscularis propria in 92 patients (98%). Kuo et al. (29) revealed that the predictive accuracy for pathologic complete response was 21.4%, 33.3%, and 53.8%, respectively, for re-biopsy, magnetic resonance imaging, and colonoscopy following chemotherapy. Lopez-Lopez et al. (30) demonstrated seventeen cases of cCR. Among the thirty-five biopsies conducted, thirty-two yielded positive results for malignancy, while the remainder were negative. Remarkably, all cCR patients exhibited negative biopsy results. Among the positive biopsies, adenocarcinoma was identified in each case, whereas among the negative biopsies, eighteen showed no malignancy, and the remaining seventeen were diagnosed with adenocarcinoma. Sixteen out of the seventeen cCR patients displayed complete pathological response, while one patient exhibited residual adenocarcinoma. Among the 50 non-cCR patients, 48 had adenocarcinoma, and two showed no malignancy. Chen et al. (31) followed 250 patients with locally advanced rectal cancer who underwent nCRT. 27.20% of patients achieved complete response, while 72.80% did not. After 100 days, biopsy as an assessment tool showed a significant increase in accuracy from 51.28% to 100%.

### 3.2 Transanal local full-thickness resection:

In recent years, transanal local excision has gained increasing attention and application in clinical practice as a surgical approach capable of removing full-thickness intestinal wall tissue. Compared to other assessment methods, transanal local excision can more accurately reflect the true state of intestinal lesions and preserve anal and rectal function during surgery. Its application is significant not only in treatment but also in the research field. For instance, Pucciarelli et al. (32) reported that in 63 patients who had undergone local excision following preoperative chemoradiotherapy for rectal cancer, 90.0% overall survival, 91.0% disease-free survival, and 96.9% local disease-free survival were respectively estimated at 91.5%, 91.0%, and 96.9%. Similarly, D’Alimonte et al. (33) demonstrated among 63 patients who underwent local excising after preoperative chemoradiotherapy for rectal cancer, overall survival was 90.0%, disease-free survival was 91.0%, and local disease-free survival was 96.9%. Furthermore, Rullier et al. (34) suggested that completion total mesorectal excision should be considered if the tumor stage was ypT2-3 in the local excision group. In its guidelines for colon and rectal surgery, Aref et al. (35) advocated for full-thickness excision after neoadjuvant therapy and demonstration of complete resection.

Most researchers advocate for a margin of approximately 1 cm around the abnormal mucosa when performing local resection. For instance, Smith et al. (36) observed lateral tumor spread beneath normal mucosa adjacent to RMAs, with extensions of up to 9 mm. However, there are contrasting viewpoints among researchers regarding the presence of residual cancerous lesions, with some arguing that they are solely deep within the abnormal mucosa of the intestinal wall. Consequently, they suggest that resection confined to the immediate vicinity of the residual lesion edge is adequate, negating the necessity to excise the surrounding mucosa of the normal intestinal wall. Local resection for rectal cancer has improved the accuracy of determining cCR and demonstrated some therapeutic efficacy. However, it faces several challenges: Local resection is limited to lesions 3-4 cm above the anal verge, as those near the verge pose risks to anal function. Healing full-thickness intestinal wall defects after radiotherapy is challenging, leading to complications such as wound dehiscence and infections, which hinder recovery. If residual tumors are detected, further surgeries may become complicated by defects and infection, potentially requiring invasive procedures like lifelong colostomy, even for patients who might have preserved anal function. Additionally, negative pathological results do not eliminate the risk of local regeneration, as factors like ulceration and scarring can complicate assessments and increase recurrence risk.

### 3.3 Radiomics technology

Conventional imaging techniques like MRI, vital for initial rectal cancer staging, have limited utility in assessing cCR after nCRT. Recent advancements in imageomics show promise for extracting high-throughput features from MRI images for cCR evaluation. Although some studies suggest imageomics can predict pCR post-nCRT, its predictive accuracy needs improvement. Currently, imageomics models remain in the research phase and lack clinical acceptance or guideline recommendations. Variability in imaging techniques and physician expertise across centers limits generalizability and reproducibility.

Based on initial computed tomography texture analysis, Vandendorpe et al. (37) developed a prognostic score that could enable more personalized treatment for each patient with localized rectal cancer, with an estimated area under the curve of 0.70. Aker et al. (38) demonstrated that among 114 patients with initial post-treatment MRI scans, the area under the curve ranged from 0.750 to 0.88 for pCR. Wen et al. (39)found that in comparison with individual radiomic models and pooled readers, a radiomic nomogram based on pre-nCRT cN stage, pre-nCRT radscore, and post-nCRT radscore achieved an AUC of 0.852. Furthermore, Feng et al. (40) demonstrated that the RAdioPathomics Integrated preDiction System (RAPIDS) had an AUC of 0.812, with favorable sensitivity, specificity, NPV, and PPV values. Additionally, The authors of Peng et al. (41) developed and validated a radiomics space-time model using machine learning for artificial intelligence interventions, achieving specificity values ranging from 0.871 to 0.983.

Radiomics, a non-invasive tool, shows promise in predicting the pCR in rectal cancer patients after nCRT. By analyzing imaging data, it enhances tumor response assessment and guides personalized treatment plans. Integrating radiomics with genomics and other omics can improve prediction accuracy and treatment efficacy while minimizing side effects. However, challenges remain. The accuracy of existing radiomics models needs enhancement, and variability due to non-standardized methods and differences in imaging techniques affects reliability. Further training and standardization are essential. Additionally, while research supports radiomics’ potential, its clinical application is still nascent, requiring large-scale studies for validation and broader implementation.

## 4 Transanal multipoint full−layer puncture biopsy

### 4.1 The current status

TMFP is vital for diagnosing rectal or colonic tumors, especially when surface biopsies are inadequate or deeper tissue evaluation is required. It plays a critical role in staging colorectal cancer by providing essential information on tumor infiltration depth into the intestinal wall. By obtaining full-thickness samples, TMFP aids in understanding disease extent, directly influencing treatment decisions such as the need for neoadjuvant therapy or the type of surgery required. In 2015, Tang et al. (42) demonstrated that the accuracy of ex vivo core needle biopsy was significantly higher than forceps biopsy (76.7% vs. 36.1%) when performed on resected specimens from 43 consecutive patients. In contrast to poor responders, the sensitivity of ex vivo core needle biopsy was lower in good responders (52.9% vs. 94.1%). Subsequently, among 16 patients demonstrating a good response to chemotherapy, eleven exhibited residual cancer cells in their final resected specimens, with four (36.4%) testing positive for cancer. In summary, routine forceps biopsy provided limited utility in detecting pathologic complete response (pCR) following neoadjuvant chemoradiotherapy (nCRT). Although core needle biopsy showed promise in identifying a subset of patients with residual cancer cells, its accuracy did not significantly improve in individuals showing a favorable response. Furthermore, a study published in the Chinese Journal of Surgery in 2023 explored the feasibility and accuracy of TMFP in assessing the residual status of cancer foci post-neoadjuvant therapy in rectal cancer patients. The study, involving 78 patients, highlighted TMFP’s high sensitivity (100% vs. 60%) and accuracy (88.5% vs. 74.4%) in detecting pathological complete response, significantly improving the determination of clinical complete response and supporting the safe implementation of the watch-and-wait strategy in rectal cancer management (43). Subsequently, another publication concentrated on developing a model based on TMFP to predict pathological complete response after neoadjuvant therapy for locally advanced rectal cancer. This study emphasizes TMFP’s predictive value in clinical settings, aiding in the more precise management of treatment approaches for rectal cancer patients. TMFP pathology demonstrated a sensitivity of 100% (45/45) and a specificity of 66.2% (43/65) in identifying pCR. The method achieved an accuracy of 80.0%, with a positive predictive value of 67.2% and a negative predictive value of 100.0% (44).

### 4.2 Development trends

TMFP’s full-thickness sampling capability allows for detailed analysis of tumor molecular and genetic characteristics, facilitating personalized therapies tailored to individual profiles. The emergence of new therapeutic agents targeting specific mutations further enhances TMFP’s utility. Concurrently, advancements in imaging technologies like high-resolution endoscopy and 3D imaging improve tumor localization and precision. Robotic-assisted techniques standardize procedures, reduce variability, and enhance surgical safety, while automated methods decrease surgical time and patient discomfort, ultimately improving efficiency in cancer care.

### 4.3 Unresolved issues

TMFP is a complex procedure requiring specialized skills and strict protocols, but the lack of uniform standards can lead to discrepancies across medical institutions, affecting detection accuracy. Additionally, variations in multi-site core needle biopsies may compromise the consistency and comparability of collected samples, making it essential to ensure they accurately represent tumor biology. Furthermore, while TMFP shows theoretical potential, its clinical applications are still nascent, necessitating large-scale studies to validate its efficacy in colorectal cancer diagnosis, treatment selection, and prognosis, as well as to compare it with traditional biopsy methods.

## 5 Current techniques for transanal multipoint full-layer puncture biopsy

TMFP is a technique designed to address the limitations of traditional diagnostic methods for rectal cancer, especially post-nCRT. This technique involves the collection of tissue samples from multiple points of the rectal wall, extending through the full thickness of the rectal tissue, including the mucosa, submucosa, and muscularis propria.

### 5.1 *In vivo* techniques

*In vivo* TMFPB techniques are performed during colonoscopy or transanal endoscopic microsurgery (TEM). These procedures allow for real-time assessment and immediate response to any complications that may arise during the biopsy.

1. Colonoscopy-Guided TMFPB: This method involves the use of a colonoscope equipped with special biopsy tools capable of reaching deeper layers of the rectal wall. It is less invasive compared to surgical methods and can be performed on an outpatient basis. However, it requires significant expertise to ensure the accuracy and safety of the procedure.
2. Transanal Endoscopic Microsurgery (TEM): TEM provides a more controlled environment for performing TMFPB. Using specialized endoscopic instruments, surgeons can precisely target areas of interest within the rectal wall. TEM is particularly useful for accessing lesions that are difficult to reach with standard colonoscopy tools.

### 5.2 Ex vivo techniques

Ex vivo TMFPB techniques are typically employed during surgical resection procedures where the rectum is removed and biopsies are taken from the excised tissue. This approach ensures a comprehensive examination of the rectal wall but is inherently more invasive and resource-intensive.

1. Specimen Mapping: After surgical resection, the rectal specimen is meticulously mapped, and multiple full-thickness biopsies are taken from pre-determined points. This method provides an extensive evaluation of the rectal tissue but is limited to patients undergoing surgery.
2. Pathological Assessment: The excised tissue undergoes detailed pathological examination to identify residual cancer cells. This technique offers the highest accuracy in detecting microscopic disease but is not applicable for patients who opt for non-surgical management strategies.

### 5.3 Ease of implementation

Implementing TMFPB techniques in clinical practice requires specialized training and equipment. Colonoscopy-guided TMFPB and TEM are less invasive and can be integrated into existing endoscopy units with proper training and investment in specialized tools. However, the precision required for these procedures demands a high level of expertise and experience.

### 5.4 Cost and resource implications

The implementation of TMFPB techniques involves significant initial costs for specialized biopsy tools and training medical staff, alongside operational expenses related to sedation and precise imaging. Post-procedure monitoring for complications adds to overall costs, but these are offset by the benefits of avoiding more invasive surgeries and their risks. Compared to traditional surgical resection, TMFPB offers a less invasive option with quicker recovery and fewer complications, potentially leading to long-term cost savings. However, careful consideration of upfront costs and resource allocation is essential for effective implementation.

## 6 Surgical technique for anterior resection of rectal cancer

### 6.1 The brief excursus of robotic surgery

Patient is positioned in lithotomy. After establish pneumoperitoneum and Trocar is inserted, the robotic bowel clamp is used to grasp the right mesorectum, pulling it ventrally and caudally. The peritoneum on the right side of the root of the sigmoid mesentery is incised, carefully dissecting the posterior rectal space while preserving the integrity of the rectal fascia propria. Dissection of lymph node group 253 between the inferior mesenteric artery and the left colic artery is performed. After fully exposing the inferior mesenteric and left colic arteries, the superior rectal artery and the sigmoid arteries are ligated, while preserving the left colic artery. The inferior mesenteric vein is also fully exposed and ligated cranially. Then the dissection laterally and cranially in Toldt’s space is extended. Dissect the continuation of the rectal fascia propria and connect it with the posterior rectal space, completing the medial-to-lateral dissection. Open the white line of Toldt, extending cranially first and then caudally. Open the peritoneum 0.5-1.0 cm above the peritoneal reflection to enter the pre-Denonvilliers’ fascia, exposing the seminal vesicles or the posterior vaginal fornix. Preserve the seminal vesicle capsule and the integrity of the Denonvilliers’ fascia. Suspend the uterus or bladder, and dissect the Denonvilliers’ fascia 0.5-1.0 cm below the seminal vesicles or posterior vaginal fornix to enter the anterior rectal space. Use the anterior and posterior rectal spaces as guides to dissect the left and right lateral ligaments of the rectum, circumferentially mobilizing the rectum. The cutting stapler to transect the rectum. Transect the mesentery of the proximal intestinal tract, transect the sigmoid colon 10 cm above the tumor, and remove the specimen. Insert the anvil head of the stapler, reinsert the proximal intestinal tract, and re-establish pneumoperitoneum. Insert the stapler through the anus, and complete the anastomosis under the robotic surgical system.

### 6.2 Discussion of the positive TMFP

In cases of positive TMFP, robotic surgery offers a minimally invasive approach that helps surgeons achieve more precise tumor removal with reduced damage to surrounding tissues, thereby increasing the likelihood of preserving the anal sphincter (45). Compared to traditional surgery, robotic surgery provides better visualization and surgical precision, aiding in sphincter protection and reducing the risk of postoperative fecal incontinence (46, 47). Therefore, robotic surgery is a safe and effective treatment option for TMFP-positive cases, improving surgical success rates and patient quality of life.

## 7 Different modalities of Total Neoadjuvant Therapy

Total neoadjuvant therapy (TNT) can be performed following three different strategies, each tailored to optimize treatment outcomes for patients with locally advanced rectal cancer: (1) Sequential Strategy: In this approach, chemotherapy is administered first, followed by radiation therapy, and then surgery. The goal is to reduce tumor size and improve the likelihood of achieving a complete pathological response before surgical intervention. (2) Concurrent Strategy: This strategy involves administering chemotherapy and radiation therapy simultaneously. The rationale is to enhance the overall therapeutic effect by combining both modalities, potentially leading to a greater reduction in tumor burden. (3) Sandwich Strategy: This involves a combination of both sequential and concurrent approaches. Initially, chemotherapy is given, followed by radiation therapy with concurrent chemotherapy, and finally, additional chemotherapy is provided after radiation before surgery.

To date, the most commonly utilized chemotherapy regimens for induction and consolidation are those based on capecitabine or 5-FU. The potential role of bevacizumab has recently been explored; however, due to limited data available, further research is warranted (48, 49). A systematic review indicated that with TNT at the beginning of induction chemotherapy, 28% of patients achieve a complete pathological response, 44% of patients experience less residual nodal disease during surgery, and 57% of patients have fewer positive surgical margins. Conversely, those receiving consolidation chemotherapy with TNT have a 90% higher likelihood of reaching a complete pathological response, although this does not enhance nodal downstaging and shows comparable rates of positive margins to standard long-course CRT. The sandwich approach of TNT has demonstrated significant efficacy in achieving pCR and substantial regression (50–52).

## 8 Conclusion

This study demonstrates the potential of transanal multipoint full-layer puncture biopsy (TMFP) to enhance the diagnosis of clinical complete response (cCR) in rectal cancer after neoadjuvant chemoradiotherapy (nCRT). TMFP improves diagnostic accuracy by collecting multi-layered tissue samples, addressing the limitations of MRI and conventional biopsies. Challenges include technique standardization and consistent sample collection. However, TMFP shows promise for personalized treatment, especially for patients considering a watch-and-wait approach post-nCRT. Future research should focus on validating TMFP through large-scale clinical trials and refining its application, possibly integrating it with other diagnostic methods for better accuracy in rectal cancer management.
